# Black Soldier Fly Larvae Meal in the Diet of Gilthead Sea Bream: Effect on Chemical and Microbiological Quality of Filets

**DOI:** 10.3389/fnut.2022.896552

**Published:** 2022-05-24

**Authors:** Marianna Oteri, Biagina Chiofalo, Giulia Maricchiolo, Giovanni Toscano, Luca Nalbone, Vittorio Lo Presti, Ambra Rita Di Rosa

**Affiliations:** ^1^Department of Veterinary Sciences, University of Messina, Messina, Italy; ^2^Institute of Biological Resources and Marine Biotechnologies, National Research Council, Messina, Italy; ^3^Department of Chemical, Biological, Pharmaceutical and Environmental Sciences, University of Messina, Messina, Italy

**Keywords:** nutritional quality, mineral profile, microbiological quality, seafood, insect meal

## Abstract

The chemical and microbiological characteristics of filets of *Spaurus aurata* L. specimens fed with diets containing a *Hermetia illucens* meal (HIM) at the 25, 35, and 50%, as a partial replacement for fish meal (FM) were evaluated. The diets, formulated to satisfy the nutritional needs of fish, were isoenergetic (22 MJ/kg gross energy), isonitrogenous (43 g/100 g, a.f.), and isolipidic (19 g/100 g, a.f.). Seventy-two specimens were randomly killed after 186 days of growing trials. Then, the filets were analyzed for chemical profile, fatty acids, amino acids, minerals, and microbial flora. Data were subjected to statistical analysis. No significant differences were observed in chemical composition. The sum of polyunsaturated fatty acids (PUFAs) showed a similar content in the filets; eicosapentaenoic acid was similar in the filets of HIM0, HIM35%, and HIM50%, whereas docosahexaenoic acid was higher in filets of the HIM0 group. n3/n6 PUFA ratio and the sum of EPA + DHA showed a high value (*p* < 0.001) in filets of the group fed with FM. No significant difference was observed in thrombogenic index and hypocholesterolaemic/hypercholesterolaemic ratio in the groups; the atherogenic index showed a higher value (*p* = 0.001) in the HIM50% group. Indispensable amino acids showed some significant (*p* < 0.0001) differences in the groups; arginine and phenylalanine content was higher in the filets of fish fed with FM; isoleucine and valine content was higher in the filets of HIM50%; leucine, lysine and methionine content was lower in the filets of HIM35%; histidine content was lower in the filets of HIM25%; tryptophan content was lower in filets of the HIM50% group. EAA/NEAA ratio showed highest value in the filets of the group that received FM. The presence of HIM in the three diets kept chromium, manganese, iron, copper, zinc, and nickel levels lower than those recommended by various authorities. Ca/P ratio showed a higher level (*p* < 0.0001) in the group fed with FM than those fed with diets containing HIM. The insect meal in the diets did not influence the microbiological profile of fish. Use of HIM as an unconventional feed ingredient in *Sparus aurata* diet looks promising, although the quality of filets may be affected.

## Introduction

The global demand and consumption of fish are increasing to meet the needs of the growing population at a faster rate than the demand for fish feed ingredients; this is leading to a rapid decline in fish meal (FM) availability and simultaneous rise in prices (1, 2). FM is the optimal animal protein source used in commercial fish feeds (3), with high bioavailability of nutrients and an adequate nutritional composition, which meets the requirements of essential amino acids and fatty acids of fish species (4, 5).

However, the use of FM is unsustainable from both environmental and economic points of view.

The aquaculture industry's most positive contribution is the search for alternative ingredients that are integrated into sustainable farming systems and provide high quality protein and lipids without negatively impacting fish health, performance, and disease resistance (6, 7), and without compromising the nutritional value of farmed fish for humans (8, 9). The use of nonconventional ingredients such as insect materials with nutritional and nutraceutical potential for human and animal health has been proposed as a relevant sustainable element of the agri-food chain (10–12). Insect meal (IM) is considered an adequate protein and lipid source that can be used as a substitute for FM in aquaculture feed because of its protein, amino acid, and fatty acid profiles (13). However, the use of insects as feed is a relatively new practice on a commercial scale, and many questions remain to be tackled: (i) the risk of transfer of pathogens into the production system (14) so much so that EFSA identifies the substrate used to feed insects as the key entry point for contamination (15); (ii) the optimal level of food replacement of FM for IM, which can vary considerably from 25 to 100% because of different compositions of larvae, fish species, and diets (16).

Among different insect species considered for possible use in aquaculture, *Hermetia illucens* is one of the most interesting because its sustainability is linked to its abilityto convert food waste materials or manure into high-quality insect nutrients (17). The proximate composition of the *H. illucens* meal (HIM) is highly variable; based on dry matter, protein and lipid contents were reported for de-fatted HIM of 47.2 and 11.8% (18) and 51.8 and 14.8% (19), respectively, and for full-fat HIM of 36.2 and 18%, respectively (20). Ash content ranged between 11 and 15% with high mineral concentrations characterized by high Ca/P ratio (21).

Although studies on the use of black soldier fly larvae meal in fish finishing started in 1987 (22), it became popular again, especially after 2017 when the European Commission allowed the use of proteins derived from 7 species of insects as alternative protein sources for aquafeed formulation (23). Previous studies have shown that replacing FM with IM in fish feeds results in changes in filet quality (24), without adversely affecting fish growth (25, 26). In particular, one problem for both producers and consumers is the consequent decrease in alfa-linolenic acid (ALA; C18:3n3), eicosapentaenoic acid (EPA; C20:5n3), and docosahexaenoic acid (DHA; C22:6n3) levels in fish filets due to the inclusion of HIM in the feed (27, 28). However, it may be possible to modify the nutritional composition of *H. illucens* through the feeding media of the insect (29–31), as observed for *H. illucens*-fed fish offal and algae where significant amounts of EPA and DHA were found (27, 32).

This study is part of a much larger research project, “Feed Insects For Aquaculture” (FIFA), which aims to reveal the nutritional value of a protein-rich insect meal (IM) produced from *H. illucens* larvae and used as a partial substitute of fish meal in *Sparus aurata* feeding. In a previous study, the proximate, fatty acid, amino acid, and mineral compositions, microbiological profile, and organoleptic characteristics of four diets formulated for *Sparus aurata* and containing 25, 35, and 50% of defatted HIM (33) as replacement for FM were characterized. Given the growing interest in HIM as an alternative protein source to replace FM in fish feeds, in another study on *Sparus aurata* fed with the diets described above, the growth performance and feed utilization efficiency were reported (not yet published), and the organoleptic properties of the filets were analyzed using a sensor-based instrument platform consisting of E-eye, E-nose with 18 MOS sensors, and a potentiometric E-tongue with 7 chemical sensors (34). This study mainly focussed on the chemical and microbiological characteristics of filets of *Spaurus aurata* L. fed with diets containing increasing levels of HIM as a partial replacement for FM.

## Materials and Methods

The experimental protocol was designed according to the Italian legislation (35) and guidelines of the current European Directive (36) on the protection of animals utilized for scientific purposes. The experimental protocol was authorized by the Italian Ministry of Health (Ministerial Authorization number 491/2019-PR released on 4 July 2019).

### Diet Formulation

All the diets were developed to meet the nutritional requirements of *Sparus aurata* and beisoenergetic (about 22 MJ/kg gross energy), isonitrogenous (about 43 g/100, as fed), and isolipidic (about 19 g/100, as fed). A control formula (HIM0) containing fish meal (FM) as an exclusive protein source of animal origin was developed. For the other three diets (HIM25, HIM35, HIM50%), the defatted *Hermetia illucens* meal was added at 25, 35, and 50% (as fed basis) to the control formula, replacing FM, to create three formulations characterized by different amounts of HIM (79, 110, and 157 g/kg). The other ingredients of the diets were adjusted to obtain iso-energetic formulas.

The diets were prepared by SPAROS Lda (Olhao, Portugal), which was commissioned to prepare the extruded fish diets. The ingredients were weighed, mixed, and grounded, and the feeds were extruded in a single screw extruder using a die diameter (d_d_) of 4 mm; after extrusion, kibbles were dried and coated with oil. The ingredients and proximate composition of the diets (HIM0, HIM25, HIM35, and HIM50%) are reported in Table 1. The fatty acid, amino acid, and mineral compositions, and microbiological profile were previously reported by Oteri et al. (33).

**Table 1:** Ingredients and proximate composition of the experimental diets.

	**DIET**			
	**HIM0**	**HIM25%**	**HIM35%**	**HIM50%**
**Ingredients, % as fed**
Fish meal	25.00	18.75	16.25	12.50
*Hermetiaillucens* meal	0	7.90	11.00	15.70
Soy protein concentrate	5.00	5.00	5.00	5.00
Wheat gluten	5.00	5.00	5.00	5.00
Corn gluten	5.00	5.00	5.00	5.00
Soybean meal 48	15.00	15.00	15.00	15.00
Rapeseed meal	5.00	5.00	5.00	5.00
Wheat meal	17.45	15.17	14.21	12.88
Whole peas	4.00	4.00	4.00	4.00
Fish oil	5.00	5.00	5.00	5.00
Rapeseed oil	10.00	9.80	9.80	9.80
Vitamin and mineral premix	1.00	1.00	1.00	1.00
Vitamin C35	0.03	0.03	0.03	0.03
Vitamin E50	0.02	0.02	0.02	0.02
Antioxidant	0.30	0.30	0.30	0.30
Sodium propionate	0.10	0.10	0.10	0.10
MCP, monocalcium phosphate	1.50	2.20	2.50	2.80
L-Lysine	0.30	0.35	0.37	0.40
L-Tryptophan	-	0.03	0.04	0.05
DL-Methionine	0.10	0.15	0.18	0.22
L-Taurine	0.20	0.20	0.20	0.20
**Proximate analysis, g/100g as fed**				
Dry matter	92.33	92.78	92.90	92.64
Crude protein	42.7	42.7	42.7	42.7
Crude fat	18.6	18.6	18.6	18.7
Crude fiber	2.3	2.2	2.2	2.1
Ash	9.3	9.3	9.4	9.3
NFE^*^	19.43	19.98	20.00	19.84

*HIM0, fish meal; HIM25, HIM35, and HIM50%, Hermetia illucens meal at the 25, 35, and 50% substitution rates of fish meal, respectively*.

* *Nitrogen-free extract, NFE (g/100g) = 100–(crude protein + crude fat + crude fiber + ash)*.

### Feeding Trial and Facilities

The experimental study on *Sparus aurata* specimens was carried out at the IRBIM facility in the CNR headquarter in Messina (Italy). On 3 February 2020, 332 fish purchased by the Ittica Caldoli Company (Lesina, Foggia, Italy) were transported to the IRBIM-CNR facility and transferred to a large tank (4.5 m^3^) for about 1 week to acclimatize to the breeding conditions. During that time, fish were fed with a commercial diet (46% protein, 16% fat; 20.7% NFE, 2.3% crude fiber; Aller Blue Omega 3 mm; Aller Aqua Company, Christiansfeld, Denmark). After 1 week of acclimation, a total of 324 mixed-sex specimens were individually weighed (average initial weight: 143.65 ± 25.94 g) and randomly divided into 12 indoor fiberglass tanks of 1.4 m^3^ (27 fish/tank, 3 replicate tanks per diet, and total of 81 fish per diet), in an open circuit system, with intake and discharge of 12 L/min of water from and to the sea (12 complete tank renewals a day). Some water parameters (pH, O_2_, temperature) were monitored daily using a professional multi-parametric probe (YSI Professional Plus Multi-Parameters Water Quality Meter probe; Xylem Inc., Yellow Springs, OH, United States). The fish were fed with the commercial diet and adapted for a further 7 days to the experimental conditions.

Twice a day (at 9:00 and 16:00 h) and 6 days a week, or for over 180 days (18 February−24th August), the fish were fed with the experimental feeds (HIM0, HIM25, HIM35, and HIM50%), initially at 0.8% and with an increase of up to 1.5% of body weight depending on water temperature. Throughout the growth trial period, tank biomass was weighed in bulk every 20 days to update daily feed ration. The tanks were inspected daily for mortality, which was, throughout the duration of the experiment,.003%.

At the end of the trial, all the fish were starved for 24 h and killed by overdose (500 mg/L) with an anesthetic (tricaine methanesulfonate solution, MS-222; Sigma-Aldrich, Italy); body weight (390 ± 49, on average) and length (28 ± 49 cm on average) were determined individually for all the fish. A subsample of 72 specimens (*n* = 18 fish per diet and 6 fish/tank) was randomly slaughtered and transported, in dry ice to the Laboratories of the Department of Veterinary Sciences-Unit of Animal Production, University of Messina (Messina, Italy), where they were gutted, fileted, deskinned, sampled in small aliquots, vacuum-packed, and freeze-dried prior to being subjected to scheduled analyses. Then, each aliquot of the filets was defrosted and homogenized with a common laboratory knife mill (Grindomix GM 200; Retsch GmbH, Haan, Germany) for the analyses described in section 2.3.

A total of 36 specimens (*n* = 9 fish per diet and 3 fish/tank) were randomly slaughtered and transported in sterile plastic bags in dry ice to the Laboratories of the Department of Veterinary Sciences–Unit of Inspection of Food of Animal Origin, University of Messina (Messina, Italy) and processed within 3 h.

### Analyses of Chemical Composition of Fish Muscle

The proximate composition of the ground filets from the four groups of fish (total number = 72; 18 fish per diet and 6 fish per replication) was determined following the AOAC (37) methods for moisture (method 950.46), crude protein (method 981.10), and ash (method 920.153).

For determination of total lipid n, the aliquots (approximately 2 g) of wet filet muscles from the four fish groups (*n* = 72) were ground, and the oil was extracted using a chloroform/methanol (2:1, v/v) solution (38). Each chemical analysis was performed in triplicate. Then, total lipids were used to prepare fatty acid methyl esters (FAMEs) for the analysis of fatty acid (FA) profile, according to the method of Christie (39). In particular, on each sample of total lipid, 2 ml of methanol:sulfuric acid (9:1, *v/v*) solution was added, and the mixture was heated at 100°C for 1 h. The FAMEs were analyzed with a Trace 1310 chromatograph (Thermo Fisher Scientific, Milan, Italy) provided with a flame ionization detector (FID). Separation of FAMEs was carried out using a 30 m × .25 mm (length × internal diameter). fused silica capillary column (Omegawax 250; Supelco, Bellefonte, PA, United States) 25-μm film, and maintained at 100°C for 5 min, from 100 to 240°C at 4 °C/min and a final isotherm of 20 min at 240°C. Injector and detector temperatures were set at 250°C. Injection volume and split ratio were 0.5 μl and 1:50, respectively. The carrier gas, helium (He), was set at a flow rate of 1 ml/min. Data acquisition and processing were performed using Chromeleon^TM^ Software (Thermo Fisher Scientific, Milan, Italy). Fatty acids of the fish samples were identified by comparing the relative retention times of FAMEs with those of a standard mix solution (mix 37 FAMEs; Supelco, Inc., Bellefonte, PA, United States) under the same analytical conditions. FA concentrations were expressed as g/100 g, where 100 g was the total of all areas of the identified FAMEs. Nutritional indices that consider different fatty acids according to their different contributions to the promotion or prevention of cardiovascular disorders were calculated from the identified fatty acids. Atherogenic (AI) and thrombogenic (TI) indices were calculated using the Ulbricht and Southgate equations (40), while hypocholesterolemic/hypercholesterolemic ratio (H/H), was calculated using the equation proposed by Santos-Silva et al. (41). The three indices were calculated as follows:


(1) $$\begin{array}{l} IA\;=\;[C12:0\;+\;(4\;\times \;C14:0)\;+\;C16:0]/(\sum^{\text{​}}n6\;-\\ \;PUFA\;+\;\sum^{\text{​}}n3-PUFA\;+\;\sum^{\text{​}}MUFA) \end{array}$$

(2) $$\begin{array}{l} \;IT\;=(C14:0\;+\;C16:0\;+\\ \;C18:0)/[(.5\;\times \;\sum^{\text{​}}MUFA)\;+(.5\times \;\sum^{\text{​}}n6-PUFA)\;+\\ \left(3\;\times \;\sum^{\text{​}}n3-PUFA)\;+\;(\sum^{\text{​}}n3--PUFA/\sum^{\text{​}}n6-PUFA)\right] \end{array}$$

(3) $$\begin{array}{l} \;H/H\;=\;(C18:1n9\;+\;C18:2n6\;+\\ C20:4n6+C18:3n3\;+\;C20:5n3\;+\;C22:5n3\;+\\ \;C22:6n3)/(C14:0\;+\;C16:0) \end{array}$$

Moreover, the peroxidation index (PI), that expresses a measure of peroxidation susceptibility and peroxidative lipid damage for a particular phospholipid membrane, was calculated using the following formula reported by Luciano et al. (42):


(4) $$\begin{array}{l} \begin{array}{c} PI\;=\;(\%dienoic\times 1)\;+\;(\%trienoic\;\times \;2)\;+ \end{array}\\ \begin{array}{c} \left(\%tetraenoic\;\times \;3)\;+\;(\%pentaenoic\;\times 4)\;+\;(\%hexaenoic\;\times 5\right) \end{array} \end{array}$$

For amino acid profile, aliquots (approximately.25 g) of wet filet muscles from the four fish groups (*n* = 72) were hydrolyzed in 10 ml of an HCl solution (6M) at 110°C for 24 h. During acid hydrolysis, asparagine and glutamine were converted to aspartic and glutamic acids (43); therefore, they were calculated as the sum of aspartic acid plus asparagine and of the glutamic acid plus glutamine. For cysteine analysis (43), an oxidation reaction using performic acid was performed for deamination prior to acid hydrolysis with an HCl solution (6M). For tryptophan analysis, acid hydrolysis was performed using 10 ml of a NaOH solution (4M) at 112°C for 16 h; then, cooling and neutralization of each sample were performed with acetic acid (44). Amino acids were analyzed with a Trace 1310 chromatograph (Thermo Fisher Scientific, Milan, Italy) provided with a flame ionization detector (FID) and a ZB-AAA Amino Acid column (10 m × 0.25 mm ID); oven temperature was programmed from 110 to 320°C at 32 °C/min, with a final isotherm of 320 °C (1 min). Injector and detector temperatures were 250 and 320°C, respectively. Injection volume and split ratio were 2.5 μl and 1:15, respectively. Procedures for purification, pre-column derivatization, and quali-quantitative analyses of each amino acid were performed using EZ:Faast Kit (Phenomenex, Torrance, CA, United States).

The mineral profile of fish samples, deprived of bones and scales, was analyzed with a high-performance dispersing instrument. About 0.5 g of the sample filets were exactly weighed in a pre-cleaned PTFE vessel by acidic wash and then digested with 7 ml of 69% Nitric acid TraceSelect and 1 ml of H_2_O_2_ at 30% (Optima™ for Ultra Trace Analysis), both purchased from Honeywell Fluka (Seelze, Germany). The closed vessels were introduced into a microwave digestion system (Ethos 1; Milestone, Bergamo, Italy) and treated with a warm-up program of 20 min at 1,000 W of microwave power. After the cooling time, the digested samples were diluted to a final volume of 25 ml with ultrapure water (resistivity 18.2 MΩ/cm) obtained from a Milli-Q Integral 3 device (Merck Millipore, Merck KGaA, Darmstadt, Germany). Samples of Mussel Tissue (CE278k) and Bovine Muscle (BB184), both from ERM (European Reference Material, Geel, Belgium), were used to verify the accuracy of the analytical procedures described above. All the solutions were stored in high-density PE bottles cleaned with a 10% (v/v) solution of HNO_3_, and were sonicated and rinsed afterward with ultrapure water. For the analysis of minerals, an ICP-OES instrument, Avio200, equipped with a vertical DualView optical system coupled with an S10 autosampler was used. Lengths of the analytical lines (nm) utilized for the analyses are reported in Table 2; the Argon line at 420.069 nm was applied as an internal standard. Table 3 reports the operational conditions of the ICP-OES. Data acquisition and processing were performed using the PerkinElmer Syngistix™ for ICP software (Perkin Elmer, Waltham, MA, United States).

**Table 2:** Analytical line length (nm) utilized for analysis.

**Element**	**nm**	**Element**	**nm**
Cr	267.716	Se	196.026
Cu	327.393	Ni	231.064
Fe	238.204	Mn	257.610
K	766.490	Zn	213.857
P	213.617	Mg	285.592
Na	589.592	Ca	317.933

**Table 3:** Operational conditions of the ICP-OES.

**Parameter**	**Conditions**
Radiofrequency power (W)	1,500
Plasma gas flow (L/min)	10.0
Auxiliary gas (L/min)	0.2
Nebulizer gas (L/min)	0.7
Sample uptake (mL/min)	1.0

Optical optimization of the ICP-OES was conducted with the procedure of the Syngistix™ software, and torch position was optimized before the analytical step using an Mn analytical line with a 1 mg/l Mn solution. The quantification of each mineral was made with external calibration curves using a set of solutions of 0.05, 0.25, 0.5, and 1 ppm arranged from a Perkin Elmer multi-element analytical solution for ICP analysis. The calibration curves for all elements were established using a calibration blank and a reagent blank, and all were found to have correlation coefficients (r^2^) ranging from 0.998 to 0.999. Detection limits (DLs) were determined by analyzing a matrix blank represented by reagents and quantities same as those used for sample preparation. Recoveries from the certified materials have reached acceptable values and higher than 85% for most of the elements and up to 95% for Zn and Cu.

### Analysis of Microbiological Profile of Fish Muscle

The analyses were carried out on samples of skin dissected from the tail to the opercula, on dorsal fleshes, and on the intestine dissected from the pylorus to the anal opening. Sampling was performed with sterile scissors and forceps by collecting the skin first from one side and the underlying flesh portion, and then repeating the operation on the opposite side and finally sampling the intestine. Each sample of skin, dorsal fleshes, and intestine was split into two aliquots and subjected to microbiological analysis. The aliquots for each sample of skin, dorsal flesh, and intestine were homogenized with buffered peptone water (Biolife, Milano, Italy, at a ratio of 1:9 w/v and with a stomacher (400 Circulator; International PBI s.p.a., Milano, Italy) for 60 s at 230 rpm and tested for the following parameters: (i) enumeration of *Enterobacteriaceae* (45) on Violet Red Bile Glucose Agar (Biolife, Milano, Italy), incubated at 37 ± 1°C for 24 h; (ii) enumeration of *Clostridium* spp. (46) on Tryptose Sulfite Cycloserine Agar (Biolife, Milano, Italy), incubated at 37 ± 1°C for 24 h under anaerobic conditions; (iii) detection of *Salmonella* spp. (47) on Chromogenic Salmonella Agar (Biolife, Milano, Italy) and Xylose Lysine Deoxycholate Agar (Biolife, Milano, Italy), both incubated at 37 ± 1°C for 24 h; (iv) detection and enumeration of *Pseudomonas* spp. on Pseudomonas Agar Base (HiMedia Laboratories, Mumbai, India), incubated at 25 ± 1 °C for 48 h; (v) detection and enumeration of *Aeromonas* spp. on GSP Agar (Pseudomonas Aeromonas Selective Agar Base) acc. to KIELWEIN (Merck, Darmstadt, Germany), incubated at 30 ± 1°C for 48 h; (vi) detection and enumeration of *Vibrio* spp. (48) on TCBS (thiosulfate citrate bile sucrose agar;bioMerieux, Marcy l'Etoile, France), incubated at 37 ± 1°C for 24 h; (vii) detection and enumeration of Specific Spoilage Organisms (SSOs) (49) on Iron Agar (Lyngby) (Oxoid Ltd., Basingstoke, Hampshire, England), incubated at 20 ± 1°C for 72 h counting black colonies as sulfide producers and white colonies as sulfide non-producers. The LODs were 10 CFU/g for the count of *Enterobacteriaceae, Aeromonas* spp., *Clostridium* spp. and SSOs, and 100 CFU/g for the count of *Pseudomonas* spp. The other aliquots for each sample of skin, dorsal flesh, and intestine were processed for the detection of *Listeria monocytogenes* (50) as follows: they were homogenized with Listeria Fraser Broth Half Concentration (Biolife, Milano, Italy), incubated at 30 ± 1°C for 20 h, followed by a passage in Listeria Fraser Broth (Biolife, Milano, Italy) incubated at 37 ± 1°C for 24 h and spread both on Agar Listeria according to Ottaviani and Agosti (Biolife, Milano, Italy) and Listeria Palcam Agar (Biolife, Milano, Italy), both incubated at 37 ± 1°C for 24–48 h.

### Statistical Analysis

For chemical composition of the filets, all the data were analyzed with the ANCOVA procedure of the XLSTAT statistical package (51). The diets (HIM0, HIM25, HIM35, and HIM50%) were used as a fixed effect and the final body weight as the covariate. In this way, the possible effects of diet and body weight have been separated. Separation of means was assessed by Tukey's test, and differences were significant if *p*<*0*.05.

To evaluate the influence of the different dietary formulations on the microbiological profile of the skin, intestine, and muscle of the fish, the microbial loads of each parameter between the different groups were compared. The normal distribution of the raw data was tested by a D'Agostino-Pearson omnibus test, and a one-way analysis of variance (ANOVA) was conducted to evaluate any significant differences between each group. A *post hoc* Tukey's test was conducted for the multiple comparisons in the obtained ANOVA data. Critical significance level (*p*) was set at 5% (0.05), and all the tests were performed two-sided. All the statistical analyses were carried out with the Graph Pad Prism 9 software (San Diego, CA, United States).

## Results

### Fish Growth Performance

In brief, at the end of the feeding trial, all the fish almost tripled their mean body weight, but there were no significant differences (*p* > 0.05) between the dietary groups for any of the considered growth performance and feed utilization efficiency indices.

### Chemical Composition of Filets

Table 4 reports the chemical composition of sea bream filet muscle. Moisture, crude protein, total lipids, and ash contents of the filets were not affected by the dietary treatments.

**Table 4:** Proximate composition (g/100 g of wet weight) of the filets of the gilthead sea bream fed with the four exerimental diets.

	**GROUP**				***p*** **-value**		**SEM^**1**^**
	**HIM0**	**HIM25%**	**HIM35%**	**HIM50%**	**D^**2**^**	**BW^**3**^**	
Fish	18	18	18	18			
Moisture	67.11	67.24	66.79	67.68	0.354	0.110	0.287
Crude Protein	20.41	19.99	20.07	19.82	0.242	0.824	0.183
Total Lipids	10.78	11.13	11.48	10.83	0.273	0.304	0.275
Ash	1.70	1.64	1.66	1.67	0.869	0.392	0.049

*HIM0, fish meal group; HIM25, HIM35, and HIM50%, Hermetia illucens meal at 25, 35, and 50% substitution rates of fish meal groups, respectively*.

*Fish: 18 per each diet, 6 fish per tank, and 3 replications per diet*.

1 *Standard error of the mean*.

2 *Diet*.

3 *Body weight*.

The fatty acid composition of the sea bream filet muscle is shown in Table 5. The saturated fatty acid, the lauric acid (C12:0) and the myristic acid (C14:0) showed significantly higher values in the HIM50% group than those observed in the fish fed fish meal and lower inclusions of *Hermetia illucens* meal. The unsaturated fatty acids, myristoleic acid (C14:1), alpha-linolenic acid (ALA, C18:2n6), and arachidonic acid (ARA, C20:4n6) showed significantly higher values in the HIM50% group than those observed in the fish fed with fish meal and lower inclusions of the *Hermetia illucens* meal; oleic acid (C18:1n9) showed a significantly higher level in the HIM25 and HIM35% groups than in the HIM50% group; eicosapentaenoic acid (EPA, C20:5n3) showed a similar content in the HIM0, HIM35 and HIM50% groups, which was significantly higher than that in the HIM25% group; docosahexaenoic acid (DHA, C22:6n3) showed a significantly higher content in the fish fed with fish meal than in the fish fed with the insect meal. The fatty acid classes of the filets are reported in Table 6. The sum of the saturated fatty acids (SFAs) showed a significantly higher value in the HIM50% group than the observed value in the HIM0 group. The sum of the monounsaturated fatty acids (MUFAs) showed a significantly lower value in the HIM50% group than the observed in the fish with fed fish meal and lower inclusions of the *Hermetia illucens* meal. The sum of the polyunsaturated fatty acids (PUFAs) and PUFAs of the n3-series showed a similar content in the HIM0, HIM35, and HIM50% groups, which was higher than that in the HIM25% group, whereas the fatty acids of the n6-series showed a significantly higher value in the HIM50% group than the observed in the fish fed with fish meal and with lower levels of inclusion of the insect meal. n3/n6 PUFA ratio (Table 6), as well as the sum of EPA+DHA, showed significantly higher levels in the fish with fed with fish meal than those of the fish fed with different inclusion of the insect meal. The indices of nutritional interest, i.e., the atherogenic (AI), thrombogenic (TI), and peroxidation (PI) indices and hypocholesterolemic/hypercholesterolemic (H/H) ratio are reported in Table 6. No significant (*p* > 0.05) difference was observed for TI and H/H in the groups, whereas the AI showed a similar level in the HIM0, HIM25, and HIM35% groups but was significantly lower and, therefore, better than that recorded in the HIM50% group. On the contrary, the PI showed a similar content in the HIM0, HIM35, and HIM50% groups but was higher than that in the HIM25% group. Body weight did not significantly (*p* > 0.05) affect the fatty acid classes, the ratio, or the nutritional indices.

**Table 5:** Fatty acid composition (g/100 g of fatty acid methyl esters)^#^ of the filets of the gilthead sea bream fed with the four exerimental diets.

	**GROUP**				***p*** **-value**		**SEM^**1**^**
	**HIM0**	**HIM25%**	**HIM35%**	**HIM50%**	**D^**2**^**	**BW^**3**^**	
Fish	18	18	18	18			
C12:0	0.05 d	0.23 c	0.40 b	0.58 a	<0.0001	0.265	0.001
C14:0	2.57 b	2.59 b	2.63 ab	2.75 a	0.001	0.636	0.012
C14:1	0.06 b	0.06 b	0.06 b	0.07 a	0.001	0.320	<0.0001
C15:0	0.20 a	0.19 b	0.19 b	0.19 b	0.001	0.690	<0.0001
C16:0	15.29	15.31	15.12	15.41	0.626	0.944	0.257
C16:1	4.17	4.16	4.25	4.16	0.610	0.716	0.031
C17:0	0.17 a	0.16 b	0.16 b	0.17 a	0.003	0.610	<0.0001
C17:1	0.03	0.03	0.03	0.03	0.296	0.407	<0.0001
C18:0	3.41	3.45	3.40	3.38	0.898	0.113	0.054
C18:1n9	40.62 ab	41.11 a	40.78 a	40.02 b	<0.0001	0.715	0.308
C18:1n7	3.11 a	3.15 a	3.06 ab	3.01 b	0.001	0.996	0.062
C18:2n6	12.83 c	12.78 c	13.17 b	13.52 a	<0.0001	0.231	0.062
C18:3n6	0.17	0.16	0.18	0.15	0.058	0.031	0.001
C18:3n3	3.26	3.28	3.25	3.30	0.661	0.348	0.010
C20:0	0.25	0.26	0.25	0.26	0.352	0.770	<0.0001
C20:1n9	2.02 a	2.02 a	1.82 b	1.79 b	<0.0001	0.339	0.006
C20:2n6	0.29 b	0.29 b	0.31 b	0.33 a	<0.0001	0.460	0.001
C20:3n6	0.16	0.17	0.17	0.17	0.505	0.112	<0.0001
C20:4n3	0.39 a	0.35 b	0.37 ab	0.36 b	0.0003	0.682	0.001
C20:4n6 ARA	0.12 b	0.12 b	0.13 ab	0.14 a	0.0002	0.224	<0.0001
C20:5n3 EPA	3.41 a	3.20 b	3.33 a	3.32 a	0.0002	0.026	0.012
C22:0	0.14	0.14	0.14	0.15	0.675	0.823	<0.0001
C22:1n9	1.12 a	0.99 b	0.93 b	0.94 b	0.0005	0.738	0.012
C22:5n3 DPA	1.28 b	1.27 b	1.34 a	1.34 a	0.047	0.677	0.006
C22:6n3 DHA	4.80 a	4.48 b	4.46 b	4.41 b	0.008	0.588	0.077

*HIM0, fish meal group; HIM25, HIM35, and HIM50%, Hermetia illucens meal at 2, 35, and 50% substitution rates of fish meal groups, respectively*.

*Fish: 18 per diet, 6 fish per tank, and 3 replications per diet*.

*#Concentration of fatty acid is expressed as g/100 g, considering 100 g the sum of the areas of all the fatty acid methyl esters (FAMEs) identified*.

*Mean values with different letters in the same row are significantly different at p < 0.05*.

*ARA, arachidonic acid; EPA, eicosapentaenoic acid; DPA, docosapentaenoic acid; DHA, docosahexaenoic acid*.

1 *Standard error of the mean*.

2 *Diet*.

3 *Body weight*.

**Table 6:** Fatty acid classes and nutritional indices of the filets of the gilthead sea bream fed with the four exerimental diets.

	**GROUP**				***p*** **-value**		**SEM^**1**^**
	**HIM0**	**HIM25%**	**HIM35%**	**HIM50%**	**D^**2**^**	**BW^**3**^**	
Fish	18	18	18	18			
SFA	22.08 b	22.33 ab	22.28 ab	22.88 a	0.042	0.619	0.469
MUFA	51.14 a	51.51 a	50.93 a	50.00 b	<0.0001	0.792	0.360
PUFA	26.72 a	26.10 b	26.72 a	27.05 a	<0.0001	0.318	0.224
n3	13.15 a	12.57 b	12.75 ab	12.74 ab	0.003	0.512	0.134
n6	13.57 c	13.52 c	13.97 b	14.31 a	<0.0001	0.380	0.071
n3/n6	0.97 a	0.93 b	0.91 bc	0.89 c	<0.0001	0.995	0.001
EPA+DHA	8.21 a	7.68 b	7.79 b	7.73 b	0.001	0.747	0.100
AI	0.33 b	0.33 b	0.34 b	0.35 a	0.001	0.941	<0.0001
TI	0.29	0.30	0.30	0.30	0.182	0.501	<0.0001
H/H ratio	3.72	3.70	3.75	3.64	0.379	0.964	0.023
PI	64.62 a	61.95 b	63.17 ab	63.30 ab	0.004	0.651	2.910

*HIM0, fish meal group; HIM25, HIM35, and HIM50%, Hermetia illucens meal at 25, 35, and 50% substitution rates of fish meal groups, respectively*.

*Fish: 18 per diet, 6 fish per tank, and 3 replications per diet*.

*SFA, sum of the saturated fatty acids; MUFA, sum of the monounsaturated fatty acids; PUFA, sum of the polyunsaturated fatty acids; EPA, eicosapentaenoic acid; DHA, docosahexaenoic acid; AI, atherogenic Index; TI, thrombogenic Index; PI, peroxidation index; H/H, hypocholesterolaemic/hypercholesterolaemic ratio. Mean values with different letters in the same row are significantly different at p < 0.05*.

1 *Standard error of the mean*.

2 *Diet*.

3 *Body weight*.

Twenty amino acids, ten indispensable amino acids (EAA), and ten dispensable ones (NEAA), were identified and quantified in the sea bream filet muscle (Table 7). Among the indispensable amino acids in the filets, arginine and phenylalanine showed significantly higher values in fish fed with fish meal than in fish fed with different inclusion of the insect meal; isoleucine and valine showed significantly higher values in fish fed with higher inclusion of the insect meal (HIM50%) than in fish fed with fish meal and with lower levels of inclusion of the insect meal; leucine, lysine and methionine showed significantly lower levels in fish of the HIM35% group, histidine in fish of the HIM25% group, and tryptophan in fish of the HIM50% group than those in the other groups. Threonine showed similar values (*p* > 0.05) in the groups. Among the dispensable amino acids, alanine showed a significantly higher level in filets of the HIM50% group than those observed in the HIM25 and HIM35% groups; aspartate + asparagine showed a significantly higher level in filets of the HIM50% group than that observed in the filets of fish fed with fish meal; glycine showed a significantly higher level in filets of the HIM50% group than that of HIM35% group; cysteine and tyrosine showed significantly higher levels in the filets of fish fed with fish meal than those of the fish fed with different inclusion of the insect meal. Hydroxylysine, hydroxyproline, and serine showed similar values in the groups. The EAA/NEAA ratio in the filets showed a significantly higher value in the fish fed with fish meal than in the fish fed with different inclusion of the insect meal. Body weight did not significantly (*p* > 0.05) affect the indispensable or the dispensable amino acid profile.

**Table 7:** Amino acid composition (g/100 g of wet weight) of the filets of the gilthead sea bream fed with the four exerimental diets.

	**GROUP**				***p*** **-value**		**SEM^**1**^**
	**HIM0**	**HIM25%**	**HIM35%**	**HIM50%**	**D^**2**^**	**BW^**3**^**	
Fish	18	18	18	18			
**Indispensable amino acids**							
Arginine	1.77 a	1.11 b	1.05 c	1.16 b	<0.001	0.295	0.014
Histidine	0.83 a	0.77 b	0.82 ab	0.86 a	<0.001	0.701	0.013
Isoleucine	1.23 b	1.25 b	1.20 b	1.34 a	<0.0001	0.404	0.014
Leucine	2.12 a	2.07 a	1.93 b	2.11 a	<0.001	0.463	0.018
Lysine	3.66 a	3.65 a	3.44 b	3.55 ab	0.011	0.236	0.047
Methionine	0.66 a	0.59 b	0.56 c	0.66 a	<0.001	0.884	0.008
Phenylalanine	1.56 a	1.16 c	1.34 b	1.04 d	<0.001	0.626	0.011
Threonine	1.10	1.18	1.16	1.16	0.212	0.691	0.027
Valine	1.15 b	1.17 b	1.13 b	1.26 a	<0.001	0.705	0.015
Tryptophan	0.02 b	0.03 a	0.03 a	0.02 c	<0.001	0.855	0.011
**Dispensable amino acids**							
Hydroxylysine	0.14	0.12	0.13	0.12	0.630	0.276	0.009
Alanine	0.96 ab	0.95 b	0.96 b	1.00 a	0.013	0.187	0.011
Aspartate+Asparigine	1.39 b	1.41 ab	1.39 ab	1.48 a	0.032	0.841	0.024
Cysteine	0.06 a	0.03 b	0.04 b	0.01 c	<0.001	0.766	0.003
Glycine	0.93 ab	0.91 ab	0.85 b	0.95 a	0.007	0.478	0.019
Glutamate+ Glutammine	0.77	0.74	0.76	0.75	0.823	0.598	0.021
Proline	0.73 a	0.72 ab	0.68 b	0.76 a	0.001	0.666	0.013
Hydroxyproline	0.33	0.33	0.34	0.34	0.456	0.193	0.008
Tyrosine	1.03 a	0.97 b	0.90 c	0.91 c	<0.001	0.889	0.014
Serine	1.07	1.15	1.14	1.09	0.058	0.615	0.024
**EAA/NEAA**	1.91 a	1.77 b	1.76 b	1.78 b	<0.001	0.206	0.018

*HIM0, fish meal group; HIM25„ HIM35, and HIM50%, Hermetia illucens meal at 25, 35 and 50% substitution rates of fish meal groups, respectively*.

*Fish: 18 per diet, 6 fish per tank, and 3 replications per diet*.

*Mean values with different letters in the same row are significantly different at p < 0.05*.

1 *Standard error of the mean*.

2 *Diet*.

3 *Body weight*.

Table 8 shows the average mineral content values in the sea bream filet muscle: macrominerals (phosphorus, sodium, potassium, calcium, and magnesium), microminerals (zinc, iron, manganese, copper, and chromium), and trace minerals (nickel). Phosphorus, showed a significantly higher value in the fish of the HIM25% group than that observed in the HIM0, HIM35, and HIM50% groups. Calcium showed a significantly higher value in the filets of the HIM0 group than those observed in the HIM50% group, whereas the HIM25 and HIM35% groups showed intermediate values. Sodium, potassium, and magnesium showed similar values (*p* > 0.05) in the groups. Due to the antagonist interaction of the Ca and P, the concentration ratio between these macroelements was calculated (52). A significantly higher level of the Ca/P ratio was observed in the HIM0 group than in the fish fed with different inclusions of *Hermetia illucens*. Among the microminerals, manganese, copper, and chromium showed similar values (*p* > 0.05) in the groups. Zinc showed a significantly lower value in fish of the HIM25 and HIM50% groups than in those of the HIM0 and HIM35% groups. Iron was significantly higher (*p* = 0.011) in the filets of the HIM0 group than in those of HIM50%, whereas intermediate values in the HIM25% and HIM35% groups were observed. The only trace mineral identified was nickel, which showed a significantly higher (*p* = 0.044) value in fish of the HIM25% group than in those of the HIM35% group; intermediate values in fish of the HIM0 and HIM50% groups were observed. Body weight did not significantly (*p* > 0.05) affect the mineral profile or the Ca/P ratio.

**Table 8:** Mineral element profile (mg/kg of wet weight) of the filets of the gilthead sea bream fed with the four exerimental diets.

	**GROUP**				***p*** **-value**		**SEM^**1**^**
	**HIM0**	**HIM25%**	**HIM35%**	**HIM50%**	**D^**2**^**	**BW^**3**^**	
Fish	18	18	18	18			
**Macrominerals**
P - Phosphorus	2,356 c	12,016 a	9,459 b	8,880 b	<0.0001	0.575	300.323
Na-Sodium	665	578	520	6340	0.254	0.652	53.05
K-Potassium	3286	3414	3503	3462	0.453	0.493	98.81
Ca-Calcium	943 a	625 ab	590 ab	484 b	0.019	0.484	96.01
Mg-Magnesium	358	344	367	356	0.709	0.526	13.83
Ca/P ratio	0.41 a	0.05 b	0.07 b	0.05 b	<0.0001	0.088	0.03
**Microminerals**
Zn-Zinc	24.96 a	17.92 b	22.36 a	16.44 b	<0.0001	0.466	2.14
Fe-Iron	5.73 a	4.09 ab	4.47 ab	3.60 b	0.011	0.281	0.41
Mn-Manganese	0.41	0.50	0.49	0.43	0.635	0.080	0.06
Cu-Copper	0.31	0.35	0.52	0.35	0.110	0.693	0.06
Cr-Chromium	0.37	0.41	0.46	0.48	0.156	0.124	0.05
Se-Selenium	0.30 a	0.22 b	0.22 b	0.35 a	<0.0001	0.377	0.02
**Trace mineral**
Ni-Nickel	1.13 ab	1.17 a	0.75 b	1.10 ab	0.044	0.094	0.101

*HIM0, fish meal group; HIM25, HIM35, and HIM50%, Hermetia illucens meal at 25, 35, and 50% substitution rates of fish meal groups, respectively*.

*Fish: 18 per diet, 6 fish per tank, and 3 replications per diet*.

*Mean values with different letters in the same row are significantly different at p < 0.05*.

1 *Standard error of the mean*.

2 *Diet*.

3 *Body weight*.

### Microbiological Profile of Filets

The results of the microbial analysis are summarized in Table 9. In the dorsal flesh of the fish from all the tested groups, no colony of the researched microorganisms was detected. No *Clostridium* spp., *Salmonella* spp., *Aeromonas*spp., *Vibrio* spp., and *L. monocytogenes* were detected in the skin and intestine of the tested specimens. The *Enterobacteriaceae* loads detected in the intestine of fish of all the groups were <2 log cfu/g, while they were not detected in the skin of any fish. SSO loads detected in the skin and intestine were <2 log cfu/g in the HIM0 and HIM25% groups but slightly higher than 2 Log cfu/g in the HIM35 and HIM50% groups although did not reach statistical significance. Regarding SSOs in the samples of the HIM0% group, only white colonies were detected while, in the samples of the HIM25, HIM35, and HIM50% groups, the white colonies compared to the black colonies accounted for majority (> 98%). *Pseudomonas* spp. was detected in the skin and intestine of all the specimens tested ( ≤ 2 log cfu/g on average). Significantly (*p* < 0.05) higher loads were observed for the *Pseudomonas* spp. in the skin of the HIM50% group compared to the HIM25% group.

**Table 9:** Microbial profile (log cfu/g) of the skin, intestine, and muscle of the gilthead sea bream fed with the four exerimental diets.

**Items**	**GROUP**											
	**HIM0%**			**HIM25%**			**HIM35%**			**HIM50%**		
	**Skin**	**Intestine**	**Muscle**	**Skin**	**Intestine**	**Muscle**	**Skin**	**Intestine**	**Muscle**	**Skin**	**Intestine**	**Muscle**
*Enterobatteriaceae*	<1	1.76	<1	<1	1.83	<1	<1	1.83	<1	<1	1.83	<1
SSOs^1^	1.90	1.93	<1	1.86	1.96	<1	2.03	2.04	<1	2.04	2.04	<1
*Pseudomonas* spp.	1.66ab	1.67	<1	1.68b	1.82	<1	1.91ab	1.87	<1	1.95a	1.93	<1
*Aeromonas* spp.	<1	<1	<1	<1	<1	<1	<1	<1	<1	<1	<1	<1
*Vibrio* spp.	<1	<1	<1	<1	<1	<1	<1	<1	<1	<1	<1	<1
*Clostridium* spp.	<1	<1	<1	<1	<1	<1	<1	<1	<1	<1	<1	<1
*L. monocytogenes*	Absent	Absent	Absent	Absent	Absent	Absent	Absent	Absent	Absent	Absent	Absent	Absent
*Salmonella* spp.	Absent	Absent	Absent	Absent	Absent	Absent	Absent	Absent	Absent	Absent	Absent	Absent

1 *Specific spoilage organisms*.

*Data reported are expressed as mean values of 9 samples analyzed (3 fish per tank and 3 replications per diet)*.

*Mean values with different letters in the same row are significantly different at p < 0.05*.

## Discussion

The diet containing insect meal did not affect the chemical composition of sea bream (*Sparus aurata*) muscle. A slight decrease in protein content was observed in fish fed with insect meal, although this variation was not statistically significant. The data are in accordance with studies on Atlantic salmons fed with a larva meal from *Hermetia illucens* (9) and sea breams fed with a larva meal from *Tenebrio molitor* (53).

Among the fatty acids in fish filets, the most interesting results were regarding the significant differences observed for some polyunsaturated fatty acids of nutritional interest. These observations are not consistent with the dogma in that differences in the fatty acid composition of muscle lipids reflect differences in dietary fatty acid contents (53, 54). In fact, despite the different *Hermetia illucens* inclusions into the diet (33) not being able to modify the fatty acid content of the feeds, the fatty acids in the sea bream muscles showed a different trend. Moreover, as observed by Sealey et al. (54), some muscle fatty acid concentrations were attenuated relative to dietary content. Among these, oleic acid (C18:1n9) ranged from 43 to 45% in the diets (33), but it only ranged from 40 to 41% in the muscles, and alfa-linolenic acid (C18:3n3) ranged from 4 to 5% in the diets (33) but was only about 3% in the muscles of all the groups. A higher concentration of DHA (C22:6n3) was detected in the muscle (4.54% on average) in comparison to its content in the diets (3.61% on average). Regarding the trend of DHA content in the muscle, significantly lower values were found in fish fed with insect meal than in those fed with the basal diet (100% fish meal), although the diets contained similar levels of DHA [Oteri et al. (33)] and were formulated to provide DHA above the estimated EFA requirements (55). The results are in agreement with the observations of Belforti et al. (8) and Pulido et al. (56). EPA appeared reduced in muscle fatty acids (3.32%, on average) compared to dietary concentrations (4.75%, on average) (33). The results agree with Sealey et al.'s observations (54), but they are not in agreement with St-Hilaire et al. (26) and Ewald et al. (28) who observed a decrease in ALA and EPA in fish fed with the inclusion of HIM in the feed. It is assumed that marine fish have a deficient capacity to bioconvert 18C precursors (C18: 2n6 and C18: 3n3) into LC-PUFAs with 20 or 22 carbon atoms such ARA, EPA, and DHA, thanks to the activity of Δ6, Δ5, and Δ4 fatty acid desaturases (57, 58). For this reason, marine fish require the presence of preformed LC-PUFAs in the diet. However, Seiliez et al. (59) demonstrated the presence and nutritional modulation of a Δ6 fatty acid desaturase in *Sparus aurata*. The same authors did not detect Δ5 and Δ4 fatty acid desaturases; the latter are responsible for the synthesis of DHA from DPA (C22: 5n3). Therefore, clear explanation and interpretation of the results obtained appear difficult, as the metabolic pathway of long-chain polyunsaturated fatty acids (LC-PUFAs) in marine fish is still under debate.

It must be mentioned tha tEPA and DHA are essential for the growth, development and health, and regulation of expression of several genes involved in lipid metabolism (60). ARA and EPA play a major role in eicosanoid production (61). The results showed that in the filets of all the experimental groups, the ARA/EPA ratio did not change (0.04), and that the levels of DHA were more depressed than those of EPA, as indicated by the changes in the EPA/DHA ratio (from 0.71 to 0.75). Moreover, Pulido et al. (56), with the aim of evaluating to what extent replacing fish meal with insect meal could alter not only the fatty acid (FA) profile of filets of *Sparus aurata* but also the FA distribution inside tryglicerides, observed that the inclusion of HIM reduced n3-PUFAs in sea bream filets but did not substantially change the presence of fatty acids important for human nutrition (e.g., EPA and DHA) in the *sn*-2 position of filet triglycerides, increasing the chances of being better assimilated and absorbed by potential consumers. The values recorded for some health lipid indices (TI: thrombogenic index and H/H: hypocholesterolemic/hypercholesterolemic ratio) appeared to be of interest, into account the contribution that each fatty acid has to influence the incidence of cardiovascular diseases (40). The similar values of these indices in the filets of all the groups suggest similar nutritional effects of all the diets on the animals. This result could be due to chitin, the main component of the exoskeleton of insects. Chitin contains high levels of chitosan with cholesterol-lowering properties in fish (62, 63), it binds with lipid micelles (cholesterol), inhibits their absorption, and increases the excretion of bile acid; thus, it interferes with the absorption of cholesterol (64). As observed by Iaconisi et al. (53), the atherogenic index showed the worst value in filets of fish fed with highest inclusion of HIM (HIM50% group), testifying a greater probability of fatty acids to affect the incidence of cardiovascular diseases (40). However, the AI and TI values observed in filets appeared much lower and, therefore, better than those reported in terrestrial animal foods (8, 65). The peroxidation index considers the contribution that PUFAs make in influencing oxidative degradation. The control of this process, causing loss of nutritional value and formation of anti-nutritional molecules, can play a central role in maintaining muscle quality (66). Lastly, the highest level of lauric acid observed in fish filets of the HIM50% group, did not affect (*p* > 0.05) fish growth performance (final body weight: 390 ± 49 g; specific growth rate: 0.75 ± 0.02). This fatty acid is dominant in black soldier fly larvae (28, 32) and is considered a bioactive compound for a possible role as an antimicrobial counteracting antibiotic resistance (67).

Based on our knowledge, this study is the first to test the effects of *Hermetia illucens* meal dietary inclusion on the amino acid composition of sea bream filets, so few comparisons with the literature are possible. On the whole, the essential amino acid profile of the fish filets reflected high protein quality. As observed by Iaconisi et al. (68), Lys and Leu were the most representative EAAs in fish filets and are the same EAAs contained at a high level in the corresponding feeds (33). Quantitatively, their content in fish filets was similar in the groups, with the exception of the muscles of the HIM35% group, although the Lys and Leu content was higher in feeds containing insect meal as a partial substitute for fish meal. One possible explanation could be that, although all the diets were formulated to be isonitrogenous, the chitin content of the feeds containing insect meal may have reduced the levels of digestible proteins or, more specifically, available amino acids (69). In fact, chitin, an unbranched N-acetylglucosamine polymer, is indigestible for many fish species that are devoid of chitinase activity (70) or with limited activity (18, 71–73). This leads to impaired digestibility of other nutrients with consequent increase in bulk, reduced feces retention time, and reduced enzyme accessibility to substrates (74). Nonetheless, the growth performance of the fish (final body weight: 390 ± 49 g; specific growth rate:.75 ± 0.02) was similar in the groups and was not affected by the dietary incorporation of HIM. Furthermore, as reported by Belghit et al. (9), *Hermetia illucens* larvae have a well-balanced EAA profile, with the exception of lysine, methionine, and tryptophan, close to that of the fish meal, considered as the protein with the best EAA profile for fish (75). In this trial, in relation to the levels of FM replacement by HIM, these amino acids were added to the diets to meet the needs of *Sparus aurata* as suggested by Magalhães et al. (76). As quantification of EAA requirements is generally based on analysis of dose-response curves with weight gain used as response criterion (77), the similar results obtained for the *in vivo* performance of the fish of all groups (final body weight: 390 ± 49 g; specific growth rate:.75 ± 0.02) demonstrate that all the diets meet the dietary amino acid requirement of *Sparus aurata*. NEAAs are not strictly necessary in the diet, because fish can synthesize them on their own; however, NEAAs can have beneficial effects on fish health and performance when present in the right concentration (78). In this study, although the diets containing the insect meal had a higher NEAA level (33) than the diet containing exclusively fish meal, the NEAA content in fish filets of all the groups was similar. This result is not in accordance with the observations of Belghit et al. (9) and could be due, as reported above, to the dietary content of chitin.

Minerals are divided into macroelements, whose needs by organisms are in large quantities, microelements, whose needs by organisms are in small quantities (79), and trace minerals typically required by organisms in such small quantities that a dietary supplement is not required (77). The functions of macrominerals include formation of skeletal structures and other hard tissues, electron transfer, regulation of acid: base equilibrium, production of membrane potentials, and osmoregulations (77). Among microelements, calcium and phosphorus are two of the major constituents of the inorganic portions of diets for fish. Quantitatively, calcium and phosphorus function primarily as structural components of hard tissues. Dietary deficiencies of most macrominerals such as calcium have been generally difficult to produce with fish species because of the presence of these ions in water (77). On the contrary, concentrations of phosphorus in natural waters are generally very low (77). Deficiency of dietary phosphorus impairs intermediate metabolism and causes reduction in fish growth and feed conversion. Integrating phosphorus into fish diets is generally more critical, because its presence in water and use by fish are limited. However, the influence of excreted phosphorus on eutrophication of receiving waters has led to a significant amount of research focused on phosphorus nutrition with the aim of minimizing phosphorus excretion (77). This appears to be of particular interest in relation to the results obtained in this study, where the phosphorus content was significantly lower in the fish filets of the control group (HIM0 = 2.4 g/kg) fed with diet with highest phosphorus content (11.45 g/kg) and formulated exclusively with fish meal (33). As reported by Rodehutscord and Pfeffer (80), phosphorus concentrations in practical dietary formulations mainly based on fish meal considerably exceed the estimation requirements. Therefore, excess in dietary phosphorus and low amount of absorbed phosphorus by fish lead to a problem of environmental impact caused by surplus phosphorus discharge into the effluents (81). However, muscle tissuess are not considered to be specific physiological sites for calcium and phosphorus (82). Phosphorus and calcium accumulate in largest amounts in bones. Borucka-JastrzeBska et al. (83) determined micro- and macroelement concentrations in different tissues of fish, and they reported that calcium distribution followed the same pattern for all three analyzed species in decreasing order: gills > muscles > skin > liver > kidney > blood. Perkowska and Protasowicki (84) showed that high levels of heavy metals were in the liver, and that the lowest ones were in the muscles. Moreover, in fish species analyzed by Roméo et al. (85), the content of metals was higher in the gills than in the muscles. Gills and liver are chosen as target organs for assessing metal accumulation. Therefore, the significantly lower values of the Ca/P ratios in fish receiving insect meal should not cause a concern. In fact, the Ca e P content did not affect anomalies in mineral homeostasis and bone mass (86). Heavy metals in the marine environment and fish contamination not only pose a threat to fish health, but by accumulating as they flow down to the natural food chain, they also pose a risk to human health (87, 88). Therefore, it is necessary to determine their content in widely consumed fish species such as *Sparus aurata*. Heavy metals such as manganese, iron, cobalt and copper are necessary for fish metabolism (89) but are toxic at high concentrations (90), while cadmium, chromium, mercury, lead and nickel are toxic metals even if present in traces in both humans and animals (91) causing numerous damages to organs (92). Although chromium is a ubiquitous metal in the environment and trivalent chromium is essential for biolife, hexavalent chromium is said to be a toxic metal with mutagenic, carcinogenic, and harmful impacts on the biota. Researchers revealed that chromium affects the physiological, behavioral, histological, biochemical, genetic, and immunological conditions of experimental organism (93). The chromium concentrations in the fish filets were found to be below the permitted level set by the European Union at 0.5 mg/kg wet weight (94). Manganese functions as a cofactor in several enzyme systems, including those involved in urea synthesis from ammonia, amino acid metabolism, fatty acid metabolism, and glucose oxidation (95). Manganese levels in fish filets were found to be below the permitted levels established by the FAO/WHO (96). Iron, essential in fish as a heme protein compound (e.g., hemoglobin, myoglobin, and cytochromes) or as a nonheme protein compound (e.g., transferrin, ferritin, and hemosiderin) (79) and for its involvement in cellular respiration both in oxidation-reduction and electron transport activity (97), also showed values below the maximum levels set by the FAO/WHO (96). Copper is an important micromineral in fish metabolism and is important for hemoglobin synthesis in many enzymatic reactions (98), but high copper concentrations can cause liver and kidney damages (99). The copper concentration determined in the fish filets of this study was below the values set by the standard regulatory limits allowed in fish samples (96). Nickel is an environmental factor that occurs at a very low level and can cause serious lung health problems such as lung cancer, fibrosis, emphysema, cancer, and kidney disease (100). In this study, nickel values were considerably lower than the permitted levels set by the FAO/WHO (96). Finally, zinc, involved in various metabolic pathways such as protein synthesis, growth, immunity, and energy metabolism in fish (79, 97) showed a significantly lower level in the fish fed with an inclusion of 25 and 50% of HIM. Overall, considering that several studies indicated that the content of these microelements in fish is influenced by surrounding water (77, 101) and by food source (102), which is the major source of elements such as iron, zinc, manganese and copper, the data would seem to highlight that the inclusion of HIM into the three diets maintains lower heavy metal levels than those recommended by various authorities (FAO, WHO, and EU).

Despite the observed differences in the lipid, protein, and mineral profiles of the filets, the organoleptic properties, in terms of color, volatile fraction, and taste, of the 4 groups of fresh filets resulted similar between the groups, suggesting that the use of HIM does not alter significantly the organoleptic properties of *Spaurus aurata* filets (34).

With regard to biological hazards, the EFSA opinion identifies the substrate used to feed the insects as the key entrance point for contamination (15); therefore, pathogenic bacteria may be present in insects depending on the substrate used and rearing conditions. Mucosal tissues, including skin and gut, are in direct contact with the environment and, thus, are the first contact points of microbes with their host representing a good control point for fish health and consumer safety (103). The reported results are confirmed, as the insect meal incorporation into the diets did not significantly influence the microbiological profile of the fish. *Pseudomonas* spp. belongs to the group of the SSOs whose loads did not differ between the various groups; therefore, although the loads of *Pseudomonas* spp. in the skin of the HIM50% group was significantly higher than in HIM25%, the detected values do not represent a relevant risk to public health. The feed can impact the microbial quality of the fish both directly, if microbiologically poor, and indirectly if residing, due to inadequate breeding set up and management, leads to modifications of water parameters (104). Therefore, we could speculate that the very low loads observed in this study are related to the good microbial quality of the feeds used, characterized only by a few SSOs with loads below 1.85 log cfu/g, as previously reported by Oteri et al. (33), as well as to the good conditions of the experimental aquaculture facility. Further studies are desirable to evaluate their application in a real scenario.

## Conclusions

This study indicates that the *Hermetia illucens* meal as a partial substitute for fish meal did not affect the proximate composition of the fish filets but significantly affected the fatty acid and amino acid profiles. However, since no detrimental effects on growth performance were found, the effects on filet quality should be considered. Furthermore, the heavy metal content and microbiological quality of the fish filets underline the safety of the *Hermetia illucens* meal as animal feed. As the price of fishmeal and fish oil increases, an economic analysis of incorporation of the *Hermetia illucens* meal into diets is needed to better assess its role as an affordable and sustainable feed in aquaculture.

## Data Availability Statement

The original contributions presented in the study are included in the article/supplementary material, further inquiries can be directed to the corresponding author.

## Ethics Statement

The animal study was reviewed and approved by Italian Ministry of Health (Ministerial Authorization Number 491/2019-PR released on date 4th July 2019).

## Author Contributions

MO: formal analysis and writing (original draft preparation and review and editing). BC: conceptualization, methodology, investigation, data curation, writing (original draft preparation and review and editing), supervision, and funding acquisition. GM: investigation and resources. GT: formal analysis. LN and VL: formal analysis and writing (original draft preparation). ADR: methodology, software, data curation, and writing (review and editing). All authors contributed to the article and approved the submitted version.

## Funding

This study was supported by the Italian Ministry of Agricultural, Food and Forestry Policies, and by European Maritime and Fisheries Fund (PO FEAMP) 2014–2020 mis. 2.47 CUP J46C18000570006, project codex 03/INA/17, Title of the project FIFA-Feed Insects for Aquaculture; Scientific Responsible: Biagina Chiofalo. The funders had no role in the design of the study; in the collection, analyses, or interpretation of data; in the writing of the manuscript, or in the decision to publish the results.

## Conflict of Interest

The authors declare that the research was conducted in the absence of any commercial or financial relationships that could be construed as a potential conflict of interest.

## Publisher's Note

All claims expressed in this article are solely those of the authors and do not necessarily represent those of their affiliated organizations, or those of the publisher, the editors and the reviewers. Any product that may be evaluated in this article, or claim that may be made by its manufacturer, is not guaranteed or endorsed by the publisher.
